# Grayken lessons: a multidisciplinary approach to care for a patient with severe ketamine use disorder

**DOI:** 10.1186/s13722-026-00645-7

**Published:** 2026-02-04

**Authors:** Maya Appley, Jessica R. Gray, Dima Abdulrahim, Owen Bowden-Jones

**Affiliations:** 1 Division of General Internal Medicine and Health Services Research, UCLA Health, Los Angeles, USA; 2 Division of General Internal Medicine, Program for Substance Use and Addiction Services, Massachusetts General Hospital, Boston, USA; 3 Central and North West London NHS Foundation Trust, Club Drug Clinic, London, UK; 4 Division of Psychology and Language Sciences, University College London, London, UK

**Keywords:** Substance use disorder, Ketamine, Club drugs, Harm reduction, Multidisciplinary care

## Abstract

**Background:**

Non-medical ketamine use is becoming increasingly common in the United States (US), but awareness remains limited among US healthcare providers.

**Case presentation:**

Here we present the case of a young woman who developed severe ketamine use disorder in the setting of an acute exacerbation of chronic post-traumatic stress disorder (PTSD). She also demonstrated symptoms concerning for two known complications of chronic ketamine use: gastrointestinal toxicity and ketamine-induced uropathy. Informed by best practices established by specialists in club drug use from the United Kingdom (UK), a multidisciplinary care plan was implemented. The plan included specialist referrals for suspected physical complications and connection with a psychiatrist and psychologist to address underlying mental health conditions driving ketamine use. With individualized, multidisciplinary support, she was able to significantly reduce ketamine use, for a period of time.

**Conclusions:**

US healthcare providers must be aware of non-medical ketamine use and associated harms, including ketamine use disorder, in order to provide effective counseling and treatment to increasing numbers of people using ketamine. In the UK, a multidisciplinary clinic was established to serve people struggling with ketamine and other club drug use, providing a model for effective, patient-centered care that can inform our approach to building systems of care for people who use ketamine and other club drugs in the US.

## Background

Ketamine is a dissociative anesthetic with amnestic, analgesic, hallucinogenic and, in low doses, stimulant properties [1]. Recently, there has also been significant interest in ketamine as a treatment for mental health conditions [2, 3]. Historically, non-medical ketamine use was rare outside of the electronic dance music scene [4], but in recent years, prevalence has risen globally [5, 6].

Though non-medical ketamine use is still relatively rare in the United States (US), rates of use have increased significantly in recent years [2, 7–11]. A recent study extrapolating data from the National Survey on Drug Use and Health (NSDUH) estimates prevalence of past year ketamine use in the US general population to have increased by 81.8% from 2015 to 2019 and 40% from 2021 to 2022 [9]. However, among adolescents in the US, the overall trend has been decreasing prevalence of ketamine use from the early 2000s to present [2, 9, 12].

Estimates of non-medical ketamine use prevalence vary, but most studies estimate prevalence of past year use to be less than 1% in the US general population [7–11]. Prevalence of lifetime use among people aged 12 and older in the US has been estimated to be 1.39% overall [2].

Prevalence of ketamine use appears to have significant regional variability in the US [13]. In one survey conducted across 17 US cities between 2021 and 2023, 1.8% of participants reported past year ketamine use. This study showed significant regional variability, with 0% of respondents in Charlotte, NC and Atlanta, GA and 12.8% of respondents in San Francisco, CA reporting past year ketamine use [13].

Ketamine use is not commonly screened for in clinical settings and it is likely that ketamine use and exposure are underreported [14]. Currently, there is limited awareness of non-medical ketamine use and associated harms, including addiction, among US healthcare providers and a lack of systems in place to effectively identify and care for people struggling with ketamine and other club drug use.

Here we present the case of a patient who experienced complications of ketamine use. We follow this with expert commentary from two specialists in ketamine and other club drug use from the UK regarding their experiences developing clinical guidance and a multidisciplinary specialty clinic for people who use club drugs. We conclude with lessons learned and implications for providers and institutions seeking to improve care for people who use ketamine and other club drugs.

### Case information

Ms. L, a 32-year-old woman with severe anxiety, depression, post-traumatic stress disorder (PTSD) and daily ketamine use, presented to a low-barrier substance use treatment clinic for assistance with ketamine cessation. Approximately nine months prior to presentation, she experienced a traumatic event and subsequent acute exacerbation of chronic PTSD symptoms. In this setting, her ketamine use escalated and she began using daily.

She first started using ketamine at raves several years prior to presentation. Until she began using daily, she had experienced no adverse consequences or issues with controlling her use and had used ketamine primarily to enhance social experiences. At presentation, her use was driven by the need to dissociate from her acute psychological distress. She most often used ketamine intranasally and occasionally rectally when experiencing nasal irritation. While she initially required less than 1 gram of ketamine daily to achieve the desired effects, at the time of presentation, her use had escalated to 5–8 grams of ketamine daily.

She noted that her use was causing significant financial stress and interfering with her ability to work and maintain relationships. For these reasons, she had attempted to stop using ketamine several times, with her longest period of abstinence being two weeks. She had not previously sought medical assistance with ketamine cessation. Within 24 hours of cessation, she would typically experience worsening of anxiety and PTSD symptoms, insomnia, restlessness, palpitations and strong cravings for ketamine, leading her to return to use. Based on this history, she was diagnosed with severe ketamine use disorder, according to the criteria outlined in the Diagnostic and Statistical Manual of Mental Disorders, Fifth Edition (DSM-5) [15].

Ms. L’s medical history was otherwise notable for chronic abdominal pain and recurrent lower urinary tract symptoms. Her abdominal pain had been attributed to gastroesophageal reflux disease, (GERD) but was not fully resolved with proton pump inhibitor therapy (PPI). She had presented to the Emergency Department several times for acute exacerbations of chronic abdominal pain. Evaluation during these presentations, including liver function tests and abdominal CT scans, were normal. She found ketamine helpful in managing chronic abdominal pain, but noted that heavy use sometimes triggered intense suprapubic and epigastric pain distinct from her typical abdominal pain. She described this pain as “K-cramps”, a known effect of ketamine gastrointestinal toxicity. Her urinary symptoms included frequency, dysuria and occasional urge incontinence. These symptoms had previously been attributed to recurrent urinary tract infections. However, urinalysis was not always consistent with infection and her symptoms were not reliably improved with antibiotics.

Previous mental health treatment for anxiety, depression and PTSD included trials of multiple medications, including several selective serotonin reuptake inhibitors (SSRIs) and bupropion, and psychotherapy. She found no significant improvement with these treatments. She had not previously been diagnosed with or participated in treatment for any substance use disorder. At the time of presentation, she was taking a low dose benzodiazepine up to three times daily as needed, as prescribed by her primary care provider (PCP) for acute anxiety. Other active substance use included weekly heavy episodic drinking, vaporized nicotine and bi-monthly cocaine and non-prescribed amphetamine/dextroamphetamine use in social settings.

After initial assessment, she was referred to the clinic’s psychiatrist and psychologist to address underlying anxiety, depression, and PTSD that appeared to be driving her ketamine use. Given her urinary symptoms, she was also referred to Urology due to concern for ketamine-induced uropathy. She was started on an atypical antipsychotic to address insomnia and anxiety. Her low-dose benzodiazepine was continued as needed. She worked with the clinic’s psychologist regularly for several months, which she found beneficial in processing her experiences and developing coping strategies for PTSD and managing ketamine cravings. With this support, she was able to reduce ketamine use to approximately once every two weeks. She desired complete cessation and considered pursuing inpatient substance use treatment to achieve this goal. However, she experienced another significant traumatic event and was subsequently lost to follow-up.

#### Expert commentary

The following commentary is provided by the following experts in the field:

1. Dr. Dima Abdulrahim, PhD, Serves as the Quality Lead for Addictions, Central and North West London NHS Foundation Trust Club Drug Clinic
2. Dr. Owen Bowden-Jones, FRCPsych MSc MBChB, Serves as a Consultant Psychiatrist, Central and North West London NHS Foundation Trust Club Drug Clinic and Honorary Professor, University College London

#### What trends are you seeing in ketamine use in the UK? What are common reasons people use ketamine?

Although less prevalent than other substances, in particular cannabis, and to a smaller extent, cocaine and MDMA/ecstasy, ketamine is relatively well-established on the non-medical drug scene in the UK, especially among younger generations [16]. Most of the ketamine available in the UK is manufactured in illicit laboratories and the diversion of pharmaceutical ketamine is very rare. It is typically sold on the illicit market as a powder or fine crystal and is most commonly used by insufflation. Data from England and Wales for the year ending in June 2022 suggest that 0.9% of people between the ages of 16 to 59 years have used ketamine in the past year, a percentage rising to 3.1% of those between the ages of 16 to 24 years. There was a marked increase in use in 2017/18, with percentages remaining largely stable since [17]. While people with ketamine use disorder comprise less than 0.1% of all people in drug treatment in England, there has been a significant increase in people seeking treatment for ketamine use in recent years. The proportion of adults seeking substance use treatment who reported difficulties with ketamine use rose from 1.6% in 2022 to 2.3% in 2024. In 2024, more than eight times as many people entered treatment for ketamine addiction than in 2014 [18].

Reasons for using ketamine often include self-medication for physical or mental health problems, including trauma. Ketamine is also used for its desired psychoactive and mind-altering effects, which, along with its lack of hangover, short duration, and relatively low cost, make it attractive to some people who use drugs. Overall, ketamine can produce a range of experiences, depending on dose. At low doses, ketamine has stimulant properties and produces distortion of time and space, visual and auditory hallucinations and mild dissociative effects. At high doses, ketamine produces more severe dissociation, known as the ‘k – hole’, where the person using ketamine experiences feelings of intense detachment and perceptions appear completely divorced from reality. The dose range required to achieve this state depends largely on the level of tolerance. For a person who is new to using ketamine, less than 1 gram could lead to significant intoxication. For a person who uses ketamine regularly, several grams could have little psychoactive effect due to tolerance [1].

Ketamine use is higher among some sub-cultures and lifestyles. These include people who frequent electronic dance music clubs and raves and men who have sex with men. Ketamine is associated with ‘clubbing’, as well as post-club ‘chill-out’, when it is used for extending the clubbing experience and as part of the drugs repertoire or stages in clubbing. Ketamine is also used by self-exploratory people and ‘psychonauts’, where high doses are deliberately used to achieve the ‘k-hole’ and hallucinogenic experiences [1].

#### What are common challenges that arise in treatment of ketamine addiction?

The main challenges which arise in treating harmful ketamine use are the ones seen with many other psychoactive substances. These include managing physical and psychological dependence, considering co-existing mental and physical health conditions and thinking about the person’s drug use in the broader family and community context. There are, however, a few things which are more specific to ketamine, one of which is so-called ‘ketamine bladder’. This is somewhat of a misnomer, as it can affect any part of the urinary tract. This is an ulcerative uropathy caused by ketamine via a mechanism that is poorly understood, but well documented. Clinical symptoms include polyuria, dysuria and hematuria and damage can be severe enough to warrant surgical intervention, including cystectomy. The management is complicated by patients using the anesthetic properties of ketamine to treat bladder pain, causing further damage to the urinary system. To break this cycle requires a careful pain management plan using alternative analgesia. Effectively addressing underlying trauma also presents a challenge and is an essential component of treating ketamine use disorder for many, as observational experience from clinical practice suggests a link between ketamine use disorder and history of trauma, especially for young women.

#### Can you tell us about why and how the Club Drug Clinic was founded?

In 2010, drug services in the UK were mostly orientated to help people using heroin and cocaine. This makes sense because these are the substances that were causing the greatest harm at the time. Young people in the UK, however, typically use other drugs and the prevalence of heroin use by young people has been falling for well over a decade. There was a clear gap in treatment provision both for people using non-opioid drugs such as ketamine, MDMA and novel psychoactive substances, as well as for younger people using drugs. The response by the National Health Service (NHS) was to establish the Club Drug Clinic in the Central and Northwest London region, a service specifically for people who use club drugs. Over the last 14 years, the service has expanded considerably due to demand and has opened satellite services in local sexual health clinics and universities. The clinic has also responded to changing trends in drug use, such as developing a specific pathway for people struggling with the use of legal and illegal prescription medications.

#### What are the essential aspects of the Club Drug Clinic model? What is the evidence for this model?

The treatment model, on paper, looks like most drug treatment services, and is grounded in a biopsychosocial approach. Treatment plans are developed based on an individual’s goals and are delivered by a multi-disciplinary team including psychiatrists, psychologists, nurses and peer mentors. What is different to many other services is the team’s cultural awareness regarding different sub-cultures and emerging drugs. Examples include a robust understanding of the cultural specifics of student populations, prescription medication use and people who engage in substance-enhanced sex (chemsex). Part of the attraction to patients of the Club Drug Clinic is knowing that the team are culturally aware with respect to particular drugs, contexts of use and sub-populations, such as people who engage in clubbing, chemsex and university students. The Club Drug Clinic also offers some treatment pathways which most UK based drug services do not. These include GHB detoxification, urological pathways, liaisons with university departments for students who are failing academically and co-location with sexual health teams to provide joint care.

The main evidence for this flexible approach is drawn from treatment outcomes, which show that the Club Drug Clinic performs very well when benchmarked against other services treating these groups in the UK. Outcomes are assessed via the National Drug and Alcohol Treatment Monitoring System (NDTMS), which includes data on self-reported substance use, treatment completion, participation in work and education and mental and physical health at 28 days and 6 months post-treatment [19]. Specific data comparing these outcomes among services are not publicly available.

#### How do patients access your services? How do you collaborate with other areas of the healthcare system?

Our service is part of the UK NHS and so is free at the point of use. Most people self-refer, although we also receive referrals from Emergency Department, mental health services, sexual health services and primary care in our locality.

We realized that some people who have challenges with substance use won’t attend substance use treatment services, as they experience this as highly stigmatizing, but may be happy to attend other health services and talk about substance use in settings they perceive as less stigmatizing. In response, we have developed satellite services in sexual health clinics and student health centers to engage people who may otherwise miss out on substance use treatment altogether. This has been a highly successful approach and we have engaged a group of people who tell us that they would have otherwise avoided addressing their substance use.

#### What are future goals for the Club Drug Clinic? If others are interested in starting similar programs, what advice would you share?

Our next goal is to spread the learning. We have already undertaken an evidence review and produced international guidance for emerging drugs, called the NEPTUNE project and free e-learning (https://www.cnwl.nhs.uk/neptune) [20]. More recently, we have also produced a free educational app for clinicians on prescription medication problems (https://addiction-to-medication.org/) [21]. Next goals are to further develop and evaluate outreach services in more universities and sexual health clinics. For those who are interested in starting similar programs, we would recommend partnering with people with lived experience of club drug use and investing in multidisciplinary partnerships with emergency services, student health, psychological services, sexual health services and urologic services.

#### What do you consider essential screening and counseling to offer patients who use ketamine?

Start by asking! Many people will not disclose use of a particular drug unless they are asked. They may be waiting to see if the clinician even knows about it and a failure to ask about a particular drug will sometimes lead the person to assume the clinician is not competent to discuss the drug. Addiction specialists should always ask directly about ketamine use when taking a thorough substance use history [1].

Screening for ketamine use in general medical settings is similar to screening for other substance use. In the US, it is recommended that adults be screened for unhealthy drug use in primary care with a validated tool, such as the Single-Question Screening Test [22], Substance Use Brief Screen (SUBS) [23], National Institute on Drug Abuse (NIDA) Quick Screen and Modified ASSIST (NM ASSIST) or Tobacco, Alcohol, Prescription Medication, and Other Substance Use Screen (TAPS) [24–27].

While there is no tool specifically designed and studied for ketamine use screening [1], some tools, such as the TAPS Tool [28] and NM ASSIST [29] explicitly ask about ketamine use if initial screening is positive and therefore may be more useful for identifying ketamine use.

If a patient screens positive for any non-prescribed substance use, including ketamine, it is an important opportunity for non-judgmental inquiry and engagement. For people who are using ketamine, it is important to screen for known adverse effects of ketamine use. We recommend inquiring about urological and gastrointestinal symptoms, cognitive changes, risks related to direct consequences of dissociation and the anesthetic effect of ketamine, including risk of accidental injury and high-risk sexual behavior, patterns and routes of use and assessing for dependency or addiction [1]. The DSM-5 criteria should be applied to assess for ketamine use disorder [30]. This information is essential for informing harm reduction counseling and treatment planning.

A variety of psychosocial interventions can be applied to management of ketamine use disorder and addiction to other club drugs, but there is no evidence to suggest that a particular psychosocial approach or type of counseling is linked to more successful outcomes than other approaches [1]. Generally, the risk level and severity of the use disorder, along with patient specific factors, including co-occurring mental health conditions, social and environmental factors, are considered when selecting psychosocial interventions. Psychosocial interventions may include, but are not limited to, brief information and interventions, motivational interviewing, mutual help groups, contingency management, cognitive behavioral therapy (CBT), CBT-based relapse prevention, mindfulness-based interventions (MBIs), acceptance and commitment therapy (ACT), dialectical behavioral therapy (DBT), psychodynamic therapy and network and environmental therapies. Relapse prevention interventions and counseling should address withdrawal symptoms such as fatigue, anxiety, dysphoria, cravings, palpitation sleep disturbances, tremulousness and delusions and any underlying mental or physical health conditions that may be driving ketamine use [1].

#### What resources do you recommend for clinicians who are interested in learning more about ketamine and other club drug use? Are there particular resources that your patients have found helpful?

For clinicians, we recommend the NEPTUNE guidance, which covers ketamine use (https://www.cnwl.nhs.uk/neptune) [20]. For patients, the main UK site for younger people using drugs is FRANK (https://talktofrank.com) [31]. In the US, San Francisco AIDS Foundation has helpful harm reduction information for ketamine use (https://www.sfaf.org/resource-library/harm-reduction-overdose-prevention-ketamine/) [32].

## Lessons learned

### Non-medical ketamine use is increasing in the US, though reasons for this remain incompletely examined

Non-medical ketamine use has been prevalent in nightclubs and electronic dance music settings for decades [4], but was very rare outside of these settings in the US until recently. Rates of past year ketamine use have been rising globally [5, 6], and in recent years, the rates of past year ketamine use appear to have risen most rapidly in the US [33]. It is unclear what is truly driving increased availability and non-medical use of ketamine, but it has been posited that this may be at least partially related to trends in medical ketamine use [2, 34]. While it has long been used clinically as an anesthetic, recently, there has been increased interest in and significant media coverage of ketamine as a promising treatment for a variety of psychiatric conditions [3, 35]. In 2019, esketamine was approved by the FDA for treatment of depression. Subsequently, demand for and prescribing of medical ketamine has increased dramatically [36].

A recent qualitative study suggests that media coverage of the medical benefits of ketamine may influence attitudes towards non-medical ketamine use among certain groups and could potentially lead to an increase in non-medical ketamine use [34]. While many people continue to use ketamine to enhance social experiences in typical settings such as nightclubs or to explore consciousness, as our experts noted, self-treatment of pain, depression, anxiety and PTSD are also increasingly common reasons that people cite for using ketamine.

While the potential relationship between expansion of medical ketamine and increasing non-medical ketamine use has not yet been studied extensively, we can draw some parallels from the approval of medical marijuana in many states over the last few decades. The relationship between medical marijuana legalization, changing attitudes and prevalence of marijuana use is complex, with many studies demonstrating conflicting findings. However, there has been an overall trend of increasing prevalence of marijuana use since medical marijuana legalization [37, 38], more significantly in states that passed medical marijuana laws [37, 39]. During this time, perceived risk of marijuana also decreased among adults [40]. Based on these trends, it is possible that we will continue to see a rise in prevalence of non-medical ketamine use as medical ketamine use becomes more common nationwide.

### Healthcare providers must be aware of non-medical ketamine use and associated harms to provide effective care

#### Effects and harms of ketamine

As shown in Table 1, ketamine has numerous potential acute and chronic effects involving multiple systems [1, 41–48]. Primary risks related to acute intoxication include accidents and injury related to ketamine’s dissociative, hallucinogenic and anesthetic effects, risk of sexually transmitted infections and overdose [1, 49, 50].

**Table 1:** Potential acute effects of ketamine and complications associated with chronic use of ketamine (adapted from Abdulrahim and Bowden-Jones 2022 [1])

	Potential acute effects of ketamine use	Potential complications of chronic ketamine use
Cardiovascular	Tachycardia	Sequelae of hypertension and tachycardia
	Hypertension	
	Chest pain	
	Palpitations	
	Brugada pattern on ECG	
	Cardiac arrest	
	Increased intracranial pressure	
Respiratory	Pulmonary edema	
	Respiratory depression	
	Respiratory arrest	
Gastrointestinal	Abdominal pain	Abdominal pain
	Nausea	Ulcers, gastritis, duodenitis
	Vomiting	Elevated liver enzymes
		Biliary dilatation
Genitourinary		Uropathy
		Nephropathy
		Erectile dysfunction
Dermatologic	Rash, transient	
Neurologic and psychiatric effects	Distortion of time and space	Cognitive impairment
	Euphoria	Depression
	Hallucinations (auditory and visual)	Delusions
	Dissociation	Psychosis
	Amnesia	Addiction
	Slurred speech	
	Dizziness	
	Numbness	
	Confusion	
	Blurred vision	
	Insomnia	
	Decreased libido	
	Cognitive impairment	
	Agitation	
	Paranoia	
	Ataxia	
	Polyneuropathy	
	Dystonia	
	Muscle rigidity, paralysis and hyperpyrexia	
	Delirium	
	Brief psychotic state or relapse of psychotic symptoms	
	Seizure	
Endocrine and metabolic	Diabetic ketoacidosis	
	Interference with antiretroviral metabolism	

While ketamine overdose is extremely rare, co-ingestion of depressants or stimulants significantly increases overdose risk due to synergistic effects [1, 46, 49, 51]. In the US, substances such as fentanyl and other depressants are frequently found to be contaminants in other drugs, especially to those that are used in powder forms, as non-medical ketamine is most often used [8]. Unanticipated contamination with fentanyl carries a high risk of fatal overdose, especially among those who are opioid naive and not using in settings with people who are prepared to respond to opioid overdose, as many people who non-medically use ketamine may be.

Chronic ketamine use is associated with several known harms that providers must be prepared to identify and address, including uropathy, nephropathy, gastrointestinal toxicity, neurotoxicity, addiction and mental health complications [1, 51]. Little data exists regarding rates of ketamine dependence and addiction among people who use non-medically. Ketamine use disorder is currently classified in the DSM-5 under phencyclidine-like substances [30]. In a qualitative study of people who were actively or previously using ketamine, 28% of respondents reported concerns related to compulsive use of ketamine [52]. In a large global survey of people who use ketamine and other club drugs who were not engaged in substance use treatment, 15.6% respondents reported more than 3 signs of dependence on ketamine [53]. When ketamine dependence or addiction does develop, it is important to identify and engage in treatment quickly to prevent further complications and harm.

Of the known complications of chronic ketamine use, gastrointestinal toxicity and uropathy are the most commonly cited [44, 48–51]. Gastrointestinal effects are experienced by approximately one third of people who use ketamine regularly and are the most common reason cited for seeking care related to ketamine use [52]. While effects including abdominal cramps, liver function test abnormalities and biliary dilatation are well documented [44, 48–51], their clinical significance in the short and long term are not well understood. People who use ketamine should be asked about abdominal pain and unexplained abdominal pain, especially in the epigastric area, should prompt screening for ketamine use.

Ketamine induced uropathy, which can affect both the lower and upper urinary tract, has been extensively documented as a prevalent and debilitating complication of chronic ketamine use [44, 45, 48–51, 54–58]. An estimated 20–30% of people who use ketamine regularly [52, 54] and up to 100% of people who use more than 5 grams of ketamine daily experience some degree of uropathy [55]. Symptoms can progress from mild irritative bladder symptoms to bladder necrosis and renal failure if ketamine exposure continues and uropathy is left untreated. The pathogenesis of ketamine induced uropathy is poorly understood, but it is thought to be related to direct toxicity of ketamine in the urine and inflammatory pathways activated by ketamine metabolism [56, 57]. Higher amounts and longer durations of use are positively correlated with higher severity of symptoms [55, 58, 59].

All patients who use ketamine should be assessed for lower urinary tract symptoms including nocturia, dysuria, increased urinary urgency and frequency, lower abdominal pain, groin pain, incontinence and hematuria and urgently referred to urologic services if there is concern for uropathy. Validated screening tools such as the International Prostate Symptom Score (IPSS) or International Consultation on Incontinence Questionnaire (ICIQ-LUTS) can be utilized in this assessment. Similarly, any patient experiencing these symptoms without a clear alternative explanation should be screened for ketamine use and referred to urologic services for further evaluation and management [58].

The initial workup for potential ketamine induced uropathy should include a thorough history of ketamine use, complete urologic history, symptom assessment with a validated tool, such as the ICIQ-LUTS or the IPSS, urinalysis with urine culture and cytology, complete blood count, complete metabolic panel, bladder scan and post-void residual measurement, ultrasound of the full urinary tract and cystoscopy with or without biopsy [58].

In their 2024 Consensus Statement on the management of ketamine induced uropathy, the British Association of Urological Surgeons conceptualizes ketamine induced uropathy in three key stages. Stage 1 is characterized by lower urinary tract symptoms related to inflammation. These symptoms typically resolve with ketamine cessation and symptomatic treatment with oral medications for overactive bladder (anticholinergic or beta 3 agonist medications), and pain (NSAIDs, neuropathic pain medications and opioids if severe). In Stage 2, structural changes, such as bladder thickening and decreased capacity, are observed. At this stage, bladder instillations and botulinum toxin-A injections may be considered after four weeks of ketamine cessation. In Stage 3, structural damage of the lower tract is more severe and the upper urinary tract may also be involved. Surgical reconstruction of the urinary tract may be required, but this is typically not considered until after six months of abstinence from ketamine [58].

#### Harm reduction for ketamine

As outlined below (see Table 2), harm reduction counseling for all people who use ketamine should include both general advice regarding overdose prevention, sexual health, safer ingestion techniques and minimizing co-ingestion, and also counseling on mitigating risks specific to ketamine use, including risks due to anesthetic, dissociative and hallucinogenic properties of ketamine and the effects of chronic use [1, 31, 32, 49, 51].

**Table 2:** Harm reduction measures for ketamine use

Potential harms	Harm reduction measures
Increased risk of accident and injury	Inquire about setting of use
	Emphasize unique risk of accident and injury due to changes in perception, anesthetic effects, dissociation and physical impairment
	Strategize with patient on harm reduction measures, such as never using alone, using with at least one sober friend, organizing safe transportation, environmental awareness such as minimizing cold/heat exposure, not using around bodies of water etc
Infection, sexual risk	Inquire about route of use (IN, IV, PO, PR), counsel and connect patient with resources for supplies for safer use as needed
	Ask about sexual practices, chemsex, strategies the patient is currently using to prevent unintended pregnancy, STI and injury
	Offer contraception, STI testing, viral infection screenings, PrEP and PEP as indicated
Overdose	Inquire about substances the patient typically uses with ketamine and ask about strategies that patient uses to moderate use
	Recommend never using alone, and “start low and go slow” approach
	Discuss synergistic effects of stimulants/depressants with ketamine and that co-use can increase risk of undesirable effects and overdose
	Consider potential contamination of ketamine with other substances, including fentanyl
	Provide naloxone, fentanyl test strips
Chronic effects	Inform patients that chronic ketamine use carries risk of uropathy, GI toxicity, cognitive impairment, depression, psychosis, dependence and addiction
	Screen for the above complications, connect to treatment as indicated

### A decentralized and multidisciplinary approach can facilitate effective, person-centered care for people who use ketamine

Multidisciplinary collaboration through multiple facets of the healthcare system is essential for engaging people who use ketamine in care, preventing and managing medical complications of ketamine use and treating addiction effectively. Our experts identify collaboration with Emergency Departments, mental health, urological services, sexual health and student health services as highly desirable partnerships for identifying and engaging people who may be struggling with ketamine use.

Referral pathways from these sites can facilitate connection to specialized addiction treatment. Providing substance use services outside of specialized addiction treatment settings, such as sexual health services and student health, can make care for people who use ketamine more accessible and reduce the barrier of stigma that prevents many people from seeking care. Within both specialized addiction treatment and general medical settings, inquiring about ketamine and other club drug use is an essential step towards reducing stigma, identifying people who may be struggling with ketamine use and helping patients feel more comfortable discussing substance use with their provider.

Psychosocial interventions conducted within a multidisciplinary team and management of underlying mental and physical health concerns driving ketamine use are the foundations of effective management of ketamine use disorder. There is no specific psychosocial modality that has been proven to be superior to others for management of ketamine use disorder [1]. The Club Drug Clinic model relies on collaboration between physicians, nurses, psychologists and peer mentors. Importantly, all professionals working within the Club Drug Clinic have knowledge and respect for cultural specifics of subgroups where ketamine use is most common, which fosters trust and engagement in care.

In both general medical settings and substance use treatment services where people who use ketamine receive care, referral pathways to specialty services to address common co-occurring disorders or complications of ketamine use are essential. Referral pathways to pain management and psychiatry are valuable, as addressing pain and mental health conditions that may be driving ketamine use is an important component of treatment for many. Referral pathways to urology are essential for expeditious connection to care for those with symptoms of ketamine induced uropathy, as delays in care can lead to serious adverse outcomes [1, 58].

While pharmacotherapy can be valuable in addressing underlying mental and physical health conditions, there are no pharmacotherapies with strong evidence for management of ketamine use disorder [60]. Successful treatment with oral naltrexone [61, 62], lamotrigine [63] and a combined regimen of intramuscular paliperidone palmitate and high dose bupropion [64] have been described in case reports. Case reports have also described effective use of benzodiazepines and antipsychotic medications for management of agitation in the setting of ketamine intoxication [42, 65] and withdrawal [66–71]. Given the limitations of the current evidence base and the rising prevalence of ketamine use, this is an important area for future exploration.

## Conclusion

Recognizing the rising prevalence of non-medical ketamine use in the US and potential for serious harms, providers must be prepared to address this emerging public health concern. Assessing provider awareness across medical specialties is a potential direction for future research to guide next steps. Further investigation into potential pharmacologic treatments and psychosocial interventions for ketamine use disorder are also needed. To inform our approach to building systems of care in the US, we can look to international models, such as the UK’s Club Drug Clinic, that have successfully implemented multidisciplinary programs and partnerships across the healthcare system to provide effective, non-stigmatizing, patient-centered care. For patients such as Ms. L, a multidisciplinary approach was essential to addressing underlying mental health concerns driving ketamine use, identifying potential medical complications and connecting with appropriate specialty care.

## Data Availability

No datasets were generated or analysed during the current study.
